# Identification of the Prodromal Symptoms and Pre-Ataxic Stage in Cerebellar Disorders: The Next Challenge

**DOI:** 10.3390/ijerph181910057

**Published:** 2021-09-24

**Authors:** Mario Manto, Aasef G. Shaikh, Hiroshi Mitoma

**Affiliations:** 1 Unité des Ataxies Cérébelleuses, Department of Neurology, Médiathèque Jean Jacquy, CHU-Charleroi, 6000 Charleroi, Belgium; 2 Department of Neurosciences, Université de Mons, 7034 Mons, Belgium; 3 Louis Stokes Cleveland VA Medical Center, University Hospitals Cleveland Medical Center, Cleveland, OH 44022, USA; axs848@case.edu; 4 Department of Medical Education, Tokyo Medical University, Tokyo 160-0023, Japan; mitoma@tokyo-med.ac.jp

**Keywords:** cerebellum, ataxias, prodromal, preataxic, cerebellar reserve

## Abstract

Cerebellar ataxias (CAs) manifest with a combination of motor incoordination, cognitive, affective and recently identified social symptoms. Novel therapies aim to stop the progression of the subgroup of the degenerative ataxias, or even to cure the disease with a functional and anatomical restoration of the cerebellar circuitry in the near future. The goal of stopping the progression of the disease is particularly relevant if applied at a very early stage of the disease, when the cerebellar reserve is only slightly impaired. Therefore, the search of the prodromal phase or pre-ataxic stage of CAs represents a very important challenge for the scientific community. The identification of pre-manifest individuals and the recruitment of individuals at risk has become a key-challenge to address neuroprotective therapies. The feasibility is high due to the recent progress in the biological and morphological biomarkers of CAs.

## 1. Turning Cerebellar Ataxias from Incurable to Treatable Diseases

Cerebellar ataxias (CAs) represent a highly heterogeneous group of neurological disorders with a core group of clinical manifestations embracing motor incoordination, cognitive and affective symptoms, and social deficits [1]. In the so-called degenerative ataxias of genetic origin which are often considered as “the typical incurable cerebellar disorders”, motor decline is progressive and irreversible. Pathological changes extend beyond the cerebellum, particularly to the brainstem and the spinal cord. Extra-cerebellar neurodegeneration causes disabilities and a reduction in life expectancy [2]. This is typically the case for spinocerebellar ataxias (SCAs), a subgroup of ataxic disorders which currently gather 48 sub-types.

The recent development of molecular therapies opens the door leading to the reversal of the irremediable course and transforms debilitating incurable diseases into treatable conditions [3]. The field of neuroprotection is also very relevant for cerebellar disorders, with the goal of stopping or slowing down disease progression.

RNA depletion or DNA addition are emerging techniques. siRNA, miRNA and CRISPR-based techniques are being validated in animal models of CAs. Antisense oligonucleotides (ASOs) represent an example of a very promising approach to revert the course of the disease even before the occurrence of symptoms [4,5]. Experimentally, the intracerebroventricular injection of ATXN3-targeting ASO into early symptomatic transgenic SCA3 mice, recapitulating the disease features of human SCA3, is associated with a reduction in polyglutamine-expanded ATXN3 and reduces the accumulation of ATXN3 after treatment [5]. Longitudinal ASO therapy rescues motor deficits, with a restoration of Purkinje neuron firing patterns. The intracerebroventricular delivery of ASO rescues the transcript levels of Kcna6 and Kcnc3, the potassium channels contributing to the homeostasy of cerebellar cortical discharges [6]. ASOs suppress aggresome formation in a human embryonic stem cell (hESC) line derived from an embryo harboring the SCA3 mutation [7].

## 2. Unravelling Early Predictors: Opening the Box of Novel Biomarkers to Complement Neuroimaging Tools

The identification of early predictors is an essential step to identify disease onset, monitor the early progression, improve our understanding of the natural history and establish novel therapies [8]. Quantitative measurements are particularly useful to extract potential biomarkers. In FXTAS, premutation carriers with FXTAS show more postural and kinetic tremor when compared with premutation carriers without FXTAS [8]. In particular, finger and hand movements show a slower speed with a loss of dexterity. In SCA2, the natural history can be subdivided into asymptomatic, prodromal, and ataxic stages (slight, moderate, and severe ataxic stages) [9]. The prodromal stage is characterized by the beginning of first motor and non-motor abnormalities without a definite manifestation of CAs [9]. In prodromal stage, SARA scores range between 0 and 2 points. Prodromal-SCA2 patients exhibit impaired gait patterns in the absence of evidence of gait impairment in SARA scale [10]. Careful ocular motor examination can reveal subtle ocular motor cerebellar deficits, which cannot be identified on the imaging studies. Namely subtle dysmetria of saccades or a change in the saccade trajectory (curved saccades) are not uncommon in early forms of cerebellar degenerative disorders. Motor adaptation testing can be a very early marker of degenerative neurological disorders. For example, a carrier of Huntington’s disease shows abnormal adaptation even in the absence of an acute disease phenotype [11]. Motor adaptation, namely saccade adaptation, can be a sensitive marker of early forms of cerebellar disorders. The test has an important caveat in that it requires a sophisticated infrastructure, and can be time-consuming to accomplish during routine clinical visits. Other neurological disorders can sometimes be early markers of cerebellar disorders. For example, REM sleep behavioral disorder can be present early on in those with multiple system atrophy (MSA). Ocular assessment includes both static and dynamic deficits which can both be used as potential biomarkers [12].

Fluid biomarkers (blood, CSF, urine, other body fluids) are used to predict the onset of the disease and to monitor the progression of ataxia. In polyglutaminopathies, the abnormal protein itself can be assessed both in blood and CSF [13]. The use of neurofilament proteins is increasing (NFL: neurofilament light chain, pNFH: phosphorylated form of heavy chain), showing a correlation with clinical rating scales [5,14,15]. For instance, serum NfL levels are increased in SCA3 and are associated with clinical disease severity, supporting serum NfL as a biomarker for disease severity in SCA3 [16]. In SCA3 knock-in mice, blood NFL increases at the presymptomatic stage. Interestingly, changes in blood concentrations are correlated with protein aggregates and early Purkinje neuron structural changes [5]. NFL could serve as biomarkers to monitor disease severity and progression. Furthermore, NFL might be a valuable tool to distinguish fast versus slow disease progressors, and thus contribute to a better stratification of patients, without the evaluation of the disease duration [5]. In ataxia-telangiectasia, there is significant correlation of NfL with age, alpha-foetoprotein, and SARA scores [17]. Numerous other fluid biomarkers are in development, including oxidative stress markers, insulin assessments, miRNA and even Tau concentrations [18,19,20]. Metabolomic studies are also being carried out. The validation of a set of fluid biomarkers in the very heterogeneous group of CAs will likely evolve in a tailored approach.

Amongst the imaging biomarkers, several important findings have appeared in the literature. Not only do brain volumetry and ROI (region of interest)-based studies allow the early identification of structural lesions, but the involvement of the white matter tracts can be demonstrated at the level of the cerebellum and cerebellar peduncles by DTI techniques [21,22,23,24]. Functional connectivity is impaired in several CAs and can be evaluated using fMRI approaches. A network-based statistics approach is promising for the understanding and follow-up of social cognition impairments in SCAs [25]. Functional/biochemical impairments can be assessed by MR spectroscopy which delineates the changes in brain metabolites including NAA (N-acetylaspartate: marker of neuronal degeneration), creatine, choline (marker of membrane turn-over), GABA/glutamate and myoinositol (glial marker). The sensitivity of MRS might be better than the sensitivity of MRI for the monitoring of the progression of spinocerebellar ataxias [26]. Volumetry outperforms clinical scores to measure disease progression in SCA1, SCA2, SCA3 and SCA7 [26]. A fixed-based analysis is a novel tractographic approach which is more sensitive than conventional DTI. Metabolic evidence of cerebral neurodegeneration is established in SCAs [27]. Techniques such as PET (positron emission tomography) and SPECT (single-photon emission computed tomography) allow the quantification of metabolic disturbances, striatal DAT binding and perfusion in cerebellar regions [28,29].

**Figure 1:**
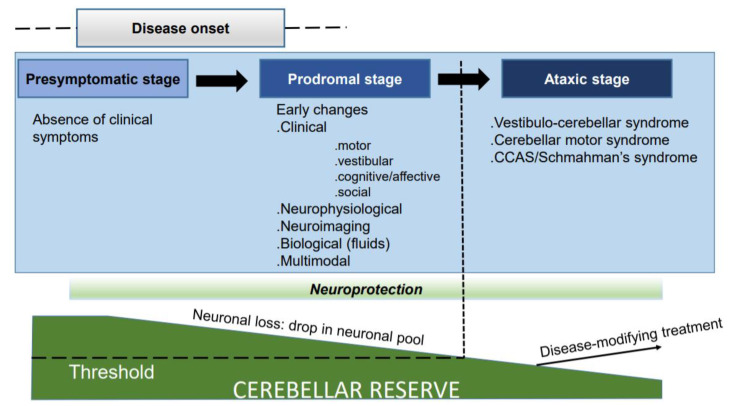
Progression of the disease from the pre-symptomatic state to the ataxic stage.

The identification of the prodromal stage is currently highly challenging. The neuronal loss leads to a decrease in the cerebellar reserve. Clinical symptoms become manifest once a threshold of manifestations is reached (Figure 1).

## 3. Preserving and Potentiating the Cerebellar Reserve

Cerebellum represents that main stock of neurons in the brain (about 60%), and therefore the question of how to preserve this “natural neuroanatomical neuronal bank” is straightforward. Cerebellar reserve is defined as the capacity for tissue compensation and restoration following pathological injury to the circuitry [30]. The anatomical organization of the cerebellar cortex into microzones, the redundant information, and the numerous forms of plasticity of the cerebellar circuitry make the cerebellum a unique machine with unique reconfiguring features [30]. The combination of abundant synaptic plasticity with the convergence of multimodal central and peripheral signals is a neurobiological substrate for compensatory and restorative mechanisms [31].

As a function of the injury, two types of cerebellar compensation and restoration can evolve: (a) structural cerebellar reserve: when the cause elicits transient structural damage in a limited area (e.g., acute events such as a stroke, traumatic injury, tumor or abscess), the lost cerebellar functions are compensated for by other cerebellar areas, which are anatomically preserved and are extra-cerebellar regions within the cerebro–cerebellar networks and brainstem-cerebellum networks; (b) functional cerebellar reserve: the neuronal loss is progressive and diffuse in this case, e.g., immune-mediated, metabolic, and degenerative CAs. Compensatory mechanisms to account for vanishing cerebellar functions are embedded in the circuitry itself and, due to the numerous inflow–outflow tracts of the cerebellum, also involve specific tracts such as the olivocerebellar pathway (climbing fibers), the ponto-cerebellar tracts (mossy fibers), the spino-cerebellar tracts (mossy fibers) and/or the aminergic/cholinergic afferent tracts. In spite of their self-recovery capacities, some patients do not show recovery from CAs. In such instances, it is assumed that the pathology has moved from a treatable condition to an untreatable state [32]. This transition is dictated by anatomical factors and the time-course of the disease. The typical example of the anatomical limitation is the acute edematous phenomenon (for instance in case of cerebellar stroke or cerebellitis) occurring in the limited space of the posterior fossa, leading to hydrocephalus and brainstem injury. Regarding the second factor, the example of immune-mediated cerebellar ataxias (IMCAs), can be used. In this group of disorders characterized by immune attacks to the circuitry, the early administration of immunotherapies at a time when the cerebellar reserve is sufficiently preserved may lead to a complete disappearance of symptoms. It is emphasized that the prompt therapeutic interventions aiming to limit irreversible neuronal loss may be challenging because neuroimaging techniques often cannot reveal cell death in the very early stages. Furthermore, specific anatomical tracts along the afferences/efferences of cerebellar paths may be involved, with difficulties in demonstrating their contribution to the clinical deficits due to the limitations of the current neuroimaging tools in terms of spatial resolution, although the advent of tractography is improving our understanding of anatomical connectivity. 

It is critical to promptly identify and address alarming symptoms that, if untreated, may lead to permanent cerebellar dysfunction. The concept gives rise to the term “brainstem attack”. The brainstem attack is a reversible deficit due to the transient loss of motor control and temporary motor, ocular motor, and vestibular deficits. A brainstem attack, classically seen in vascular compromise, i.e., the posterior circulation of transient ischemic attacks, can have an important differential in impending autoimmune disease that, if untreated, can lead to permanent damage, as is the case with its vascular etiology. The vascular brainstem attack symptoms should be promptly addressed with aggressive antiplatelet therapy, which is the adequate perfusion pressure maintenance, and, if needed, the addition of antilipid agents. Immune etiologies, on the contrary, are addressed with immunomodulators, steroids, or intravenous immunoglobulin therapy. There are distinct differences between vascular and immune brainstem attacks; vascular attacks lead to episodes of several hours to days in length, while immune etiology is generally associated with much prolonged, days- to week-long deficits. If untreated, the impairment in the immune etiology leads to a neurodegenerative disorder, while vascular etiology may result in a stroke. Typically occurring in a stroke, the brain stem attack can also present with a neurodegenerative or autoimmune disease. The brainstem attack is thought to be secondary to the brainstem or cerebellar Purkinje neuron dysfunction. These neurons provide critical feedback to a number of motor circuits, including facilitating saccades, pursuit, gaze holding, and VOR.

In terms of cerebellar reserve, the process of neuronal death resulting in atrophy helps us to better understand how neuronal loss manifests clinically and why some patients develop cerebellar symptoms quickly despite a small apparent atrophy. In a subgroup of patients, the atrophy of the cerebellum is silent whereas in others the symptomatology remains unchanged. This reflects the collapse of the dynamic capacity for compensation and restoration, mainly due to advanced cell death [33]. Reserve can be grasped as a moderator between pathological changes and outcome and is closely dependent on ageing [34,35].

## 4. Neuromodulation to Restore Cerebellar Function

Novel tools are now available to restore cerebellar functions in a non-invasive way, especially by means of neuromodulation techniques. The cerebellum represents an ideal target for this approach for several reasons, including the anatomical location of the cerebellum immediately below the skull, the very high concentration of neurons in the cerebellar cortex, its organization into modules linking the cerebellar cortex/nuclei/the inferior olive, the multiple forms of plasticity between parallel fibers/Purkinje neurons, the remote effects of the cerebellum upon the brainstem nuclei/basal ganglia/cerebral cortex, the multiple roles of the cerebellum in oculomotor and motor controls, cognitive operations, and social/affective regulation [36]. The neuromodulation of the cerebellum can be applied in the field of motor control (essential tremor, dystonia, Parkinson’s disease, cerebellar disorders) and in a focal lesions like stroke. This modality can be useful in a large range of neuropsychiatric disorders for which a dysfunction or lesions of the cerebellum are presumed (schizophrenia, bipolar disorders, major depressive disorders, and generalized anxiety disorder). Yet, this conceptually attractive technique requires more research. The cerebellar circuit is complex, and there can be a substantial intersubject variability. Rigorous and large studies are necessary to determine the most appropriate neuromodulation parameters. When neuromodulation techniques facilitate cerebellar control on other motor and cognitive/affective centers, the degree of damage in the target region should be also considered. Again, understanding the network re-organization is critical.

One of the challenges of the cerebellum community is to identify the prodromal stages of cerebellar disorders, so that early therapeutic strategies can be administered at a time when the cerebellar reserve is still in a position to respond. It is however, encouraging that currently irreversible and incurable cerebellar degenerative disorders will soon be addressable. The ongoing cutting-edge research in neurotherapeutics, neuromodulation, and physiological and biological marker development will bring us closer to a much more effective treatment early on in the disease course.

## 5. Conclusions

The identification of pre-manifest individuals and the recruitment of individuals at risk has become a major challenge to address neuroprotective strategies. Due to the rarity of CAs, their clinical heterogeneity, and the variability in penetrance and clinical progression, multi-center studies are required. There is space for improvements in clinical rating scales, because the currently used scales show both floor and ceiling effects, which create difficulties in handling patients with very mild deficits, in addition to the subjective effect inherent to clinical scores. A combination of clinical scales with quality-of-life measures, wearable sensors allowing free-living motor activity monitoring, neuroimaging biomarkers (MRI, MR spectroscopy, DTI) and fluid biomarkers (blood, CSF) is likely the best approach, not only to identify conversions from the asymptomatic stage to symptomatic condition, but also for the general monitoring of ataxias [37,38]. Pure clinical tools, such as the evaluation of the body mass index should not be excluded, as shown recently for BMI, which is significantly decreased in SCA2, with an association with age at onset and progression rate [39]. We may also have to re-consider how trials are designed in patients’ registries of CAs given the major heterogeneity in clinical profiles and progression.

## Data Availability

The concepts discussed in this article are not based on raw data.
